# Drug repurposing in cardiovascular inflammation: Successes, failures, and future opportunities

**DOI:** 10.3389/fphar.2022.1046406

**Published:** 2022-10-21

**Authors:** Laura Chaffey, Annabell Roberti, David R. Greaves

**Affiliations:** Sir William Dunn School of Pathology, University of Oxford, Oxford, United Kingdom

**Keywords:** inflammation, drug repurposing, cardiovascular disease, drug delivery, drug reengineering, chrono-pharmacology

## Abstract

Drug repurposing is an attractive, pragmatic approach to drug discovery that has yielded success across medical fields over the years. The use of existing medicines for novel indications enables dramatically reduced development costs and timescales compared with *de novo* drug discovery and is therefore a promising strategy in cardiovascular disease, where new drug approvals lag significantly behind that of other fields. Extensive evidence from pre-clinical and clinical studies show that chronic inflammation is a driver of pathology in cardiovascular disease, and many efforts have been made to target cardiovascular inflammation therapeutically. This approach has been met with significant challenges however, namely off-target effects associated with broad-spectrum immunosuppression, particularly in long-term conditions such as cardiovascular disease. Nevertheless, multiple anti-inflammatory medicines have been assessed for efficacy in cardiovascular clinical trials, with most of these being repurposed from their original indications in autoimmune conditions like rheumatoid arthritis. In this review, we discuss the mixed successes of clinical trials investigating anti-inflammatory drugs in cardiovascular disease, with examples such as anti-cytokine monoclonal antibodies, colchicine, and methotrexate. Looking to the future, we highlight potential new directions for drug repurposing in cardiovascular inflammation, including the emerging concepts of drug re-engineering and chrono-pharmacology.

## 1 Introduction

The repurposing of existing medicines for novel indications is a concept that has been around since the very idea of using medicines to treat disease. Throughout history, treatments found to be effective for one particular ailment have been applied in other contexts due to either shared symptoms across diseases, or as a result of observations from patients already given the treatment. A prominent example of this historical form of “drug repurposing” is aspirin. Aspirin has been used for centuries in the form of Willow bark to treat a range of conditions from back pain to fever to headaches, which proved effective due to their shared inflammatory a etiology. Since 1897, aspirin has been used in its purified form for many of these same inflammatory conditions, and in 1971 Vane and colleagues delineated the molecular mechanism for this activity *via* the inhibition of cyclooxygenase (COX) and production of prostaglandins (Montinari et al., 2019). Alongside its anti-inflammatory effects, the additional anti-thrombotic activity attributed to aspirin has now seen it repurposed to become one of the most widely used drugs for prevention of cardiovascular disease (CVD) to this day.

Drug repurposing has several advantages over *de novo* drug development strategies. Foremost is the financial benefit to repurposing an existing medicine. The average cost of bringing a brand-new drug to market has been estimated at US$2-3bn (Nosengo, 2016), with a large portion of this cost ascribed to extensive studies of pharmacokinetics and safety testing. Repurposing a drug for a novel indication costs just a fraction of that however, at an average of US$300m (Nosengo, 2016), as early stage testing is not needed. Moreover, repurposed drugs take on average 6.5 years to be approved for their new indication, whereas *de novo* drugs typically have development pipelines spanning 10–15 years, with many candidates failing in early safety and toxicity trials. Drug repurposing therefore offers a relatively low-risk, pragmatic strategy for pharmaceutical companies and these economic incentives have led to increased interest and financial investment in drug repurposing programmes over the past decade or so. This notion was brought to the forefront during the COVID-19 pandemic, when repurposing of medicines such as Dexamethasone (Recovery Collaborative Group, 2021) allowed the unmet clinical need of patients with severe COVID-19 to be addressed far more rapidly than could be achieved otherwise.

Historically, most cases of drug repurposing have been serendipitous, typically resulting from epidemiological data gathered on side effect profiles of drugs from patients already taking them. A prime example of this approach is sildenafil. Originally developed to combat hypertension, sildenafil has since been successfully repurposed for the treatment of erectile dysfunction after observations made during clinical trials. More recently, drug repurposing approaches have become strategic, with high throughput phenotypic cell screening and computational modelling of drug-target interactions between structurally similar compounds being commonly used methods (reviewed in Roberti et al., 2022).

Despite CVD remaining the leading cause of death worldwide, new drug approvals for cardiovascular indications lag significantly behind those of other fields and the rate of new approvals has been steadily declining. For instance, new drug approvals by the U.S Food and Drug Administration (FDA) for cardiovascular indications declined from 16% of total approvals in 1997 to just 2% in 2018 (Zannad et al., 2021). Although a “productivity crisis” has been declared across the drug development landscape (Pammolli et al., 2011), this trend does not carry across to other fields such as oncology where approvals have instead increased from 16% to 27% of total approvals over the same period (Zannad et al., 2021). One proposed explanation for this is the difficulty of conducting cardiovascular clinical trials (Fordyce et al., 2015; Zannad et al., 2021; Abdelsayed et al., 2022). Generally, trial regulators require “hard” end-points like cardiovascular events including MI and stroke, rather than biomarker or surrogate outcomes such as levels of low density lipoprotein or C-reactive protein. As these events are relatively infrequent in comparison to equivalent end points used in fields such as oncology, and particularly so in patients already receiving standard care e.g. statins or ACE inhibitors, large patient cohorts and long follow-up periods are needed in order to achieve statistical power in such trials. Together, this makes cardiovascular trials both difficult and, more pertinently, expensive to run (Fordyce et al., 2015; Zannad et al., 2021; Abdelsayed et al., 2022). Drug repurposing and its aforementioned financial advantages could therefore be a particularly useful approach in cardiovascular medicine.

**FIGURE 1:**
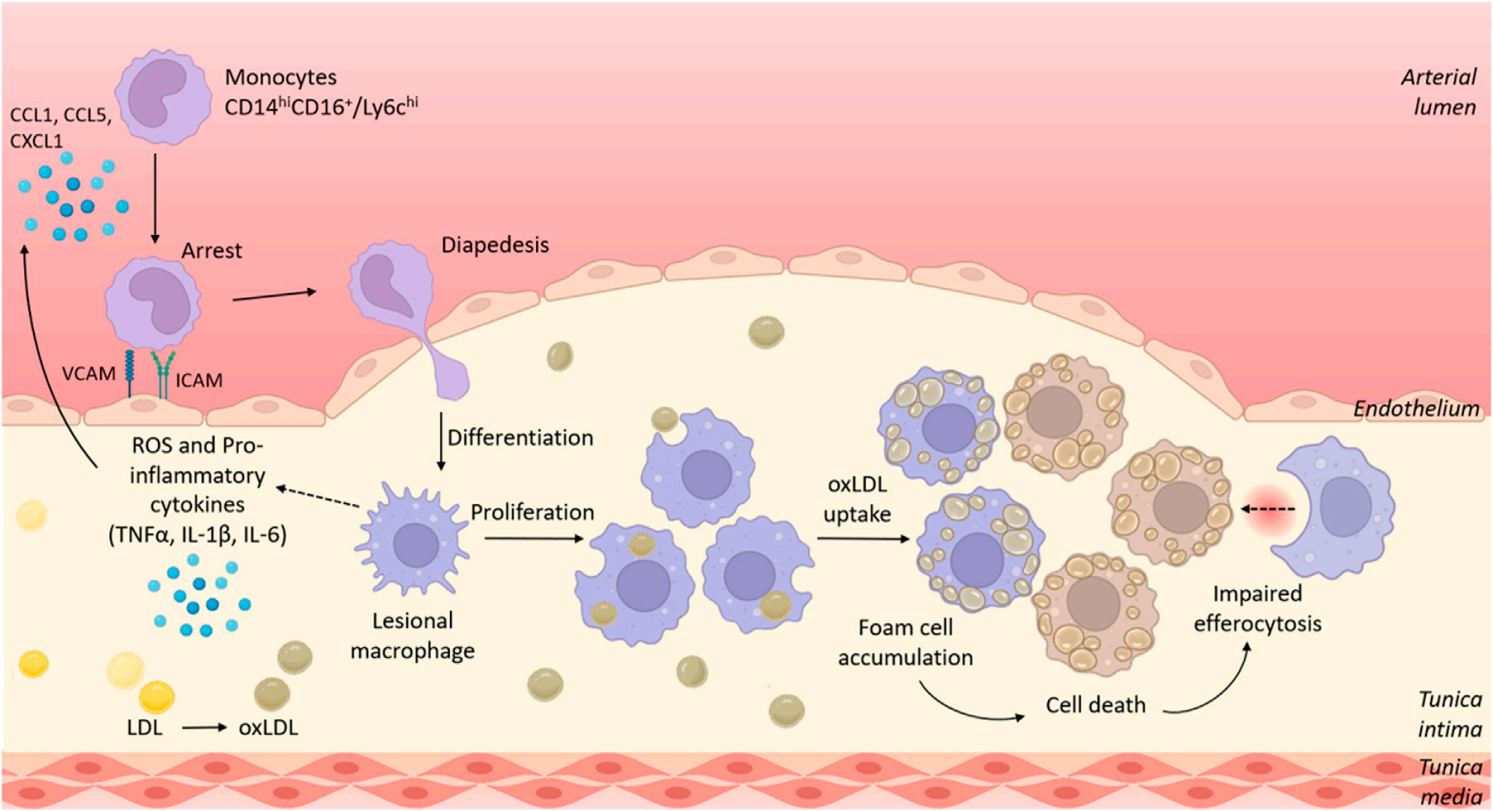
The role of myeloid cells in the development of atherosclerotic lesions. Circulating murine Ly6c^hi^/human CD14^hi^CD16+ monocytes are recruited to sites of atherosclerotic plaques/fatty streaks *via* signalling of chemokines such as CCL2, CCL5 and CXCL1. Recruited monocytes adhere to the activated endothelium that overexpresses adhesion molecules including VCAM-1 and ICAM-1. Monocytes transmigrate through the endothelium where they differentiate into macrophages. Initially monocyte recruitment, and later local proliferation of lesional macrophages contribute to growing plaque size. Within the plaque, macrophages adopt a pro-inflammatory phenotype, resulting in production of reactive oxygen species (ROS) and pro-inflammatory cytokines such as TNFα, IL-1β and IL-6. Lesional macrophages phagocytose oxidised lipoproteins *via* scavenger receptors such as CD36 and become lipid-laden foam cells. Dying foam cells accumulate within the plaque to form a necrotic core in part because of impaired cell clearance *via* efferocytosis. Figure generated using BioRender.

Inflammation is a driver of CVD pathogenesis. Since 1997, a correlation has been identified between inflammatory marker C-reactive protein (CRP) and cardiovascular risk in patients regardless of blood lipid levels (Ridker et al., 1997), and increased risk of CVD has long been noted in patients with existing inflammatory conditions like rheumatoid arthritis (RA) (Hansildaar et al., 2021; Agca et al., 2022; Conrad et al., 2022). Furthermore, although current treatments for CVD primarily aim to reduce circulating lipid levels, some of the efficacy of statins has been attributed to their anti-inflammatory action (Ridker, 2009). These observations are supported by overwhelming data from *in vitro* studies and animal models of CVD to show that inflammatory cells are key to atherogenesis, the pathophysiological process underlying most CVD cases (Figure 1). Cells of myeloid origin including monocytes and macrophages have been heavily implicated in pathological inflammation in atherosclerosis and make attractive therapeutic targets due to phenotypic plasticity between M1-like cells that are pro-inflammatory and M2-like cells that promote tissue repair and inflammation resolution. Consequently, many attempts have been made to therapeutically target the inflammatory compartment of CVD with varied levels of success.

In this review, we reflect upon findings from cardiovascular clinical trials of anti-inflammatory medicines, discuss current challenges in repurposing anti-inflammatory drugs for CVD, and suggest avenues that may be explored in the future to overcome these.

## 2 Clinical findings from targeting inflammation in cardiovascular disease

In this section, we review findings from clinical studies that have sought to treat CVD by directly targeting inflammation. Owing to their exploratory nature and the common pathways involved across different inflammatory disease contexts, these trials are often drug repurposing trials, and many of the interventions tested have been safely used to treat inflammatory conditions including gout and RA for decades. A summary of cardiovascular trials studying repurposed anti-inflammatory drugs can be found in Table 1.

**TABLE 1:** Cardiovascular Clinical Trials of anti-inflammatory therapeutics.

Pharmacological Agent	Biological target	Start date	Patient population	Intervention	Key findings	References, Trial identifier
Anakinra(MRC-ILA Heart Study)	IL-1β	2007	NSTEMI within 48 h (n = 182)	Placebo vs. anakinra 100 mg s.c daily for 14 days	49% reduction in CRP levels over first 7 days Significant increase in MACE at 1 year follow up	Morton et al. (2015) 2006–001767–31
Anakinra	IL-1 β	2008	STEMI within 24 h (n = 10,30,99)	Placebo vs. anakinra 100 mg s.c. Daily or twice daily for 14 days	No difference in LV end systolic volume index or LV function Significant reduction in CRP for 14 days of treatment	Abbate et al. (2020) NCT00789724, NCT01175018, NCT01950299
Canakinumab (CANTOS)	IL-1β	2011	previous MI CRP ≥ 2 mg/L (n = 10,061)	Placebo vs. canakinumab 50/150/300 mg s.c every 3 months	15% reduction in MACE in 150 mg group only. No difference in blood lipids. Adverse effects include neutropenia, increased death due to infection or sepsis	Ridker et al. (2017) NCT01327846
Tocilizumab (ASSAIL-MI)	IL-6	2017	STEMI within 6 h (n = 199)	Placebo vs. tocilizumab 280 mg single dose i.v	5.6% increase in myocardial salvage index No significant difference in infarct size at 6 months Significant reduction in CRP	Broch et al. (2021) NCT03004703
Ziltivekimab* (RESCUE)	IL-6	2019	Chronic kidney disease,CRP ≥ 2 mg/L (n = 264)	Placebo vs. ziltivekimab 7.5/15/30 mg every 4 weeks for 24 weeks, s.c	Significantly greater reduction in CRP from baseline at 12 weeks in all ziltivekimab groups vs. placebo (77%, 88%, 92% vs. 4%) Significant reduction in lipoproteinA, but no change in LDL:HDL ratio	Ridker et al. (2021) NCT03926117
Etanercept (RENEWAL)	TNFα	1999	Chronic heart failure (n = 2048)	Placebo vs. etanercept 25 mg weekly/twice weekly, s.c	Trials terminated early due to lack of benefit No significant difference in clinical status, hospitalisation for heart failure or CV death, at 24 weeks	Mann et al. (2004)
Infliximab (ATTACH)	TNFα	2000	Chronic heart failure (n = 150)	Placebo vs. infliximab 5/10 mg/kg at 0, 2, 6 weeks	No significant difference in clinical score at 14 or 28 weeks follow up Increased incidence of MACE in 10 mg/kg infliximab group at 28 weeks TNF levels initially reduced by then significantly increased above baseline up to 28 weeks in both infliximab groups	Chung et al. (2003)
Colchicine (LoDoCo)	cytoskeletal rearrangement, non-specific NLRP3 inflammasome	2008	Coronary artery disease (n = 352)	Untreated vs. colchicine 0.5 mg daily, p.o	Significant reduction in incidence of acute coronary syndrome, cardiac arrest, stroke	Nidorf et al. (2013) ACTRN12610000293066
Colchicine (COLCHICINE-PCI)	cytoskeletal rearrangement, non-specific NLRP3 inflammasome	2013	Patients referred for PCI (n = 713)	Placebo vs. 1.2 mg colchicine pre coronary angiography, then 0.6 mg pre-PCI, p.o	No significant difference in PCI-related myocardial injury or MACE within 30 days Elevation of plasma IL-6 and CRP from baseline - 24 h s significantly reduced in colchicine group Increased adverse gastrointestinal symptoms in colchicine group	Shah et al. (2020) NCT02594111
Colchicine (LoDoCo2)	cytoskeletal rearrangement, non-specific NLRP3 inflammasome	2014	Coronary artery disease (n = 5,522)	Placebo vs. colchicine 0.5 mg daily, p.o	31% reduction in MACE	Nidorf et al. (2020) ACTRN12614000093684
Colchicine (COLCOT)	cytoskeletal rearrangement, non-specific NLRP3 inflammasome	2015	MI within 30 days (n = 4,445)	Placebo vs. colchicine 0.5 mg daily, p.o	Significant reduction in MACE in colchicine group No significant difference in incidence of infection, but increased risk of pneumonia with colchicine	Tardif et al. (2019) NCT02551094
Colchicine (COPS)	cytoskeletal rearrangement, non-specific NLRP3 inflammasome	2015	Acute coronary syndrome (n = 795)	Placebo vs. colchicine 0.5 mg twice daily for 1 month, then daily for 11 months, p.o	No significant difference in MACE Increased total death incidence in colchicine group, mostly related to sepsis	Tong et al. (2020a)ACTRN12615000861550
Colchicine (COVERT-MI)	cytoskeletal rearrangement, non-specific NLRP3 inflammasome	2018	STEMI within 12 h (n = 192)	Placebo vs. colchicine 2 mg p.o, then colchicine 0.5 mg twice daily, p.o for 5 days	No significant reduction in infarct size at 5 days No significant reduction in MACE at 3 months follow up No difference in inflammatory marker (WBC count, CRP) at 48 h	Mewton et al. (2021) NCT03156816
Methotrexate (METIS)	T cell activation, adenosine signalling	2007	Ischemic chronic heart failure (n = 50)	Placebo vs. methotrexate 7.5 mg weekly, p.o for 12 weeks	No difference in 6 min walk time before vs. after treatment No effect on CRP levels No effect on incidence of MACE	Moreira et al. (2009) NCT00759811
Methotrexate (CIRT)	T cell activation, adenosine signalling	2013	Previous MI or coronary artery disease, plus T2D (n = 4,768)	Placebo vs. methotrexate 5–20 mg weekly, p.o	Trial terminated early due to lack of benefit No difference in incidence of MACE No difference in inflammatory markers (WBC count, CRP, IL-1β, IL-6) at 8 months Increased incidence of leukopenia, non-basal-cell skin cancer	Ridker et al. (2019) NCT01594333
Methotrexate (TETHYS)	T cell activation, adenosine signalling	2013	STEMI within 12 h (*n* = 84)	Placebo vs. methotrexate 0.05 mg/kg for 6 h	No effect on creatine kinase release over first 72 h No difference in CRP levels Significantly worse LVEF in methotrexate group at 3 months No effect on incidence of MACE	Moreira et al. (2017) NCT01741558
Cyclosporine (CIRCUS)	T cell activation, macrophage ROS/cytokine production	2011	STEMI within 12 h (*n* = 970)	Placebo vs. cyclosporine A 2.5 mg/kg, i.v	No effect on any cause death No effect on LV remodelling or MACE incidence at 6 months	Cung et al. (2015) NCT01502774
Cyclosporine (CYCLE)	T cell activation, macrophage ROS/cytokine production	2012	STEMI within 6 h (*n* = 203)	Placebo vs. cyclosporine A 2.5 mg/kg, i.v	No effect on ST-segment resolution at 1 h No effect on LV remodelling or incidence of MACE at 6 months	Ottani et al. (2016) NCT01650662
Allopurinol (ALL-HEART)	xanthine oxidase	2013	Ischemic Heart Disease (*n* = 5,215 target)	Placebo vs. allopurinol 600 mg daily, p.o	No effect on incidence of CV events	Makenzie, (2022) ISRCTN32017426
Aldesleukin (LILACS)	IL-2 receptor Treg cells	2017	Ischaemic heart disease (*n* = 25) and NSTEMI (*n* = 16)	Placebo vs. aldesleukin 0.3–3 × 10^6^ (IU) daily for 5 days, s.c	Increase in Tregs but not CD4^+^ effector T cells	Zhao et al. (2020) NCT03113773

* ziltivekimab has been developed specifically for cardiovascular disease and has not yet gained regulatory approval. NSTEMI, non-ST segment elevation myocardial infarction; STEMI, ST segment elevation myocardial infarction; s.c, subcutaneous; CRP, C-reactive protein; MACE, major adverse cardiovascular event; LV, left-ventricular; MI, myocardial infarction; CV, cardiovascular; i.v, intravenous; p.o, per os; WBC, white blood cell; T2D, Type 2 Diabetes; LVEF, left ventricular ejection fraction; ROS, reactive oxygen species

### 2.1 Cytokine inhibiting therapies

#### 2.1.1 IL-1β inhibitors

To date, the most notable trial to target inflammation in CVD was the landmark Canakinumab Anti-Inflammatory Thrombosis Outcomes Study (CANTOS) in which interleukin 1 (IL-1β) signalling was targeted (Ridker et al., 2017). Prior to CANTOS, which began recruitment in 2011, IL-1β had been implicated in atherogenesis through numerous pre-clinical studies *via* a variety of mechanisms involving several cell types including pro-inflammatory programming of endothelial cells (ECs), increased immune cell trafficking *via* induction ICAM-1, VCAM-1, and CCL2, proliferation of smooth muscle cells (SMCs) *via* induction of platelet derived growth factor (PDGF), and activation of monocytes and macrophages (van Tassell et al., 2013; Szekely and Arbel, 2018). CANTOS became a pivotal study for the field and was touted as the key to either proving or disproving the then controversial “inflammatory hypothesis” of atherosclerosis; the notion that inflammation and not lipid accumulation alone plays a causal role in atherogenesis. The preceding 20 years had provided vast amounts of evidence that inflammation contributed to atherosclerosis, however until CANTOS this had not been directly addressed in the clinic. The chosen IL-1β inhibitor, canakinumab, had been used since its FDA approval in 2009 for the treatment of the rare inherited disorder cryopyrin-associated periodic syndromes (CAPS), in which IL-1β is over-secreted, as well as for the treatment of other inflammatory conditions such as gout and periodic fever syndromes.

In CANTOS, 10,061 patients who had a history of MI and clinically elevated levels of CRP, were randomised into groups receiving regular subcutaneous injections of either placebo or canakinumab at 50, 150, or 300 mg. It was found that patients receiving canakinumab had reduced incidence of major adverse CV events (defined as MI, stroke, and CV related death) compared with placebo, although this difference was a mild 15% reduction and only statistically significant for the 150 mg group. Importantly for the “inflammatory hypothesis”, this reduction was found to be independent of lowering circulating lipid levels, suggesting that lipids are not the sole driver of atherosclerosis progression in the patient cohort studied. Furthermore, the greatest benefit was seen in patients who achieved a substantial reduction in CRP, once again supporting the notion that systemic inflammation has a causal role in atherogenesis and CVD (Ridker et al., 2017).

Despite these results being comparatively modest to those seen with lipid lowering statins and PCSK9 inhibitors (Cheung et al., 2004), and although these findings have not yet translated into any changes in clinical care, CANTOS was widely regarded as a success. The study has undoubtably paved the way for future clinical studies into the inhibition of inflammatory signalling as a therapeutic approach for chronic CVD. On the other hand, canakinumab was not without the adverse effects expected of a potent anti-inflammatory drug, with treatment causing increased infections due to neutropenia, as well as a significantly increased incidence of death due to infection or sepsis, although this did not affect overall death rates (Ridker et al., 2017). Notably, the IL-1 receptor antagonist anakinra has failed to improve outcomes in cardiovascular trials (Morton et al., 2015; Abbate et al., 2020).

Regardless of efficacy, the feasibility of using canakinumab, a fully human monoclonal antibody therapeutic for a chronic clinical application such as atherosclerosis has been questioned. One 150 mg vial of canakinumab, which is currently marketed by Novartis, costs just under £10,000 (National Institute for Health and Care Excellence, 2022) and given that there are an estimated 2.3 million people living with coronary heart disease (CHD) in the United Kingdom (British Heart Foundation, 2022), this is simply not a viable therapeutic solution and clearly negates the cost benefits of a drug repurposing approach. Indeed, a cost-effectiveness analysis of canakinumab in CVD using data from CANTOS estimated that canakinumab cost $6.4 million USD (£5.3 million GBP) per quality-adjusted life year (QALY) gained (Sehested et al., 2019), whereas the threshold for cost efficacy set by the National Institute for Health and Care Excellence (NICE) in the United Kingdom is typically £20,000 per QALY (Ogden, 2017).

#### 2.1.2 IL-6 inhibitors

Despite financial concerns over using biologics, other single-cytokine inhibiting monoclonal antibodies have been assessed in CVD. ASSAIL-MI was a small study of 199 patients which aimed to determine the effects of IL-6 inhibition in patients following ST-segment elevation MI (STEMI) (Broch et al., 2021), as IL-6 levels have been correlated with both infarct size and prognosis post-MI (Ritschel et al., 2014). The study found that treatment with the anti-IL-6 therapeutic antibody tocilizumab, which binds and blocks the IL-6 receptor, significantly improved the primary trial outcome of myocardial salvage index compared to placebo, yet it had no significant effect on infarct size at 6 months follow up. Interestingly, pre-clinical studies have provided evidence to suggest that early IL-6 signalling immediately following MI is likely to have cardioprotective properties (Fontes et al., 2016), possibly explaining lack of benefit observed in trial patients who were given tocilizumab within 6 h of symptoms onset. In the future it may be of interest to examine the effects of delayed intervention with tocilizumab in MI once IL-6 signalling is initiated, as experimental evidence suggests that only prolonged IL-6 signalling becomes deleterious (Fontes et al., 2016). Novo Nordisk are currently developing a novel IL-6 inhibiting monoclonal antibody specifically for cardiovascular indications. The antibody, ziltivekimab, has so far been effective at reducing CRP levels in patients with chronic kidney disease, who are at increased risk of developing CVD and are contraindicated for alternative therapies such as colchicine (Ridker et al., 2021). Ziltivekimab’s effect on incidence of cardiovascular events is currently being investigated in a Phase III trial (ZEUS; NCT05021835).

#### 2.1.3 TNFα inhibitors

TNFα signalling and its role in the progression of cardiovascular disease has been studied extensively however animal studies have found conflicting effects of TNFα inhibition (reviewed in Rolski and Błyszczuk, 2020). Nevertheless, due to its key role in chronic inflammation and heavily influenced by the success of TNFα inhibition in autoimmune conditions such as RA (McKellar et al., 2009), TNFα inhibitors were among the first biologics to be clinically investigated for CVD. Between 1999 and 2001, the RECOVER and RENAISSANCE trials investigated the effects of subcutaneous etanercept in a total of 2048 patients with chronic heart failure (CHF). The combined analyses of these trials (termed RENEWAL) concluded that etanercept showed no benefit with no difference in incidence of death or hospitalisation for CHF at 24 weeks, causing both trials to be terminated early due to lack of efficacy (Mann et al., 2004).

In a separate trial of CHF in 150 patients (ATTACH), TNFα inhibitor infliximab was given intravenously at 5 or 10 mg/kg doses over a 6 week period. Similarly to RENEWAL, it was found that infliximab treatment had no significant effect on clinical status at follow up 14 or 28 weeks after intervention, although serious adverse events including death or hospitalisation for HF were increased in the 10 mg/kg infliximab group at 28 weeks follow up. Interestingly in this trial, analysis of blood markers of inflammation showed that although treatment initially reduced TNFα, levels were raised above baseline by 28 weeks follow up in both infliximab groups although other inflammatory markers CRP and IL-6 remained significantly reduced up to 14 weeks. This effect could suggest induction of a compensatory TNFα mechanism, which may be partially responsible for the adverse effects seen in the 10 mg/kg group. Curiously, meta-analyses of RA patients show that although anti-TNFα therapy does not affect incidence of MI in itself, patients who respond to TNFα therapy have significantly reduced MI risk compared to those who do not respond (Dixon et al., 2007). Patients with RA have increased risk of CVD (McKellar et al., 2009), likely owing to their systemic inflammation. Overall, the disappointing results of the above trials suggest that single cytokine inhibition, although useful in helping us to decipher the contribution of individual cytokines to CVD pathogenesis, is not likely to be a successful therapeutic strategy in the complex multicellular processes of post-MI recovery and chronic CVD.

### 2.2 Colchicine

A perhaps more pragmatic alternative repurposing candidate that fulfils criteria of being cheap and widely available is colchicine, an inexpensive plant-derived medicine that dates back to 1500BC when it was used by ancient Egyptians. Colchicine is a standard treatment for gout and familial Mediterranean fever and its immunomodulatory effects are achieved by inhibiting tubulin polymerisation, thereby impeding cellular processes that involve cytoskeletal rearrangement (Deftereos et al., 2022). Although this effect is not cell-type specific, neutrophils are disproportionately affected by colchicine due to their lack of P-glycoprotein membrane efflux pump. This results in accumulation of intracellular colchicine, leading to impaired neutrophil functionality and in particular, reduced ability to achieve chemotaxis that is heavily dependent on cytoskeletal rearrangement (Deftereos et al., 2022). Additionally, the NLRP3 inflammasome is believed to be inhibited by colchicine (Demidowich et al., 2016). Inflammasome activation and subsequent IL-1β production *via* caspase 1 activity has been a pharmacological target of great interest in CVD in recent years as inflammasome activation contributes to tissue damage and disease progression (Tong et al., 2020), and genetic knockout of inflammasome components results in overall reduced plaque burden in murine models of atherosclerosis (Karasawa and Takahashi, 2017). Despite having reduced specificity, such pleiotropic effects of colchicine may provide advantage over single cytokine inhibiting therapies with reduced chance for compensatory mechanisms to be triggered.

Colchicine has been assessed in several clinical trials for cardiovascular disease (recently reviewed in Deftereos et al., 2022) and has largely been found to have positive effects. The COLCOT study of 4,445 patients found a significant reduction in adverse CV events in post-MI patients receiving daily colchicine *versus* those receiving placebo (Tardif et al., 2019). Similarly, the LoDoCo and LoDoCo2 trials found colchicine reduced incidence of CV events in 6,054 patients with chronic coronary disease (Nidorf et al., 2013, 2020). However, two additional trials in patients with acute coronary syndrome (COPS) and in patients with STEMI (COVERT-MI) reported no benefit to taking colchicine (Tong et al., 2020; Mewton et al., 2021). Notably, in these trials colchicine treatment correlated with adverse immunosuppressive effects such as an increased risk of pneumonia (in COLCOT) and increased rate of all-cause death (in COPS), largely owing to increased incidence of sepsis. These unfortunate adverse effects are not surprising and are indicative of the inherent challenges of inhibiting inflammation, yet may be overlooked if overall disease burden is sufficiently reduced with treatment. It should also be noted that colchicine has toxicity-associated adverse effects, particularly gastrointestinal symptoms, with which comes a particularly narrow therapeutic window. This may also present an additional challenge to its repurposing.

Interestingly, a secondary analysis of COLCOT data performed by Samuel et al. (2021) estimated that over the two-year trial period, per-patient costs were 47% lower for patients receiving colchicine compared to those receiving only standard care (at $265 *versus* $502 CAD) and over a lifetime period of 20 years this difference was increased to 69%. Overall, they reported that colchicine increased quality-adjusted life years (QALYs) from 8.82 to 11.68, demonstrating the potential of colchicine therapy going forward if issues with immunosuppression and toxicity can be addressed.

### 2.3 Methotrexate

Despite promising results in pre-clinical studies, some anti-inflammatory medicines have been less successful in CVD trials. The Cardiovascular Inflammation Reduction Trial (CIRT) was run in parallel to CANTOS and aimed to assess the efficacy of methotrexate in CVD (Ridker et al., 2019). The trial was conceived based upon clinical observations that RA patients who were taking low dose methotrexate had reduced incidence of major CV events (Choi et al., 2002; Micha et al., 2011). In addition to its use as a chemotherapeutic agent, methotrexate has been used in the treatment of RA since the 1980s, making it an early example of drug repurposing success. Despite this long history, methotrexate’s activity in inflammatory disease is not well understood. Possible mechanisms by which methotrexate may exert its anti-inflammatory effects are *via* the inhibition of cytokine production through blocking T cell activation, and enhancing signalling *via* adenosine receptors (Chan and Cronstein, 2002). Similarly to colchicine, these multiple biological effects of methotrexate may be advantageous to treatment of CVD.

In CIRT, 4,768 patients with previous MI or coronary disease, as well as type 2 diabetes (T2D) or metabolic syndrome were randomised into groups receiving methotrexate or placebo. No difference in the primary endpoint of major CV event was seen between the two groups, and additionally methotrexate was found to have no effect on inflammatory markers such as CRP, IL-1β, IL-6 or total white blood cell count at 8 months follow up (Ridker et al., 2019). Additional trials with smaller patient cohorts have also failed to see benefit of methotrexate in chronic CVD (Moreira et al., 2009, 2017).

### 2.4 Cyclosporine

Cyclosporine is an immunosuppressive agent used to prevent immune rejection after organ transplantation and was thought to be a good candidate for drug repurposing in CVD due to its multifaceted anti-inflammatory modes of action. Cyclosporine is a calcineurin inhibitor that inhibits activity of T cells and myeloid cells *via* inhibition of the essential transcription factor NFAT (Liddicoat and Lavelle, 2019). However, in two separate trials of patients experiencing STEMI, cyclosporine failed to show beneficial effect on ST-segment resolution, LV remodelling or incidence of CV events within the trial periods of 6 months and 1 year respectively (Cung et al., 2015; Ottani et al., 2016).

### 2.5 Summary of clinical trials

The above findings demonstrate the frustrations faced by targeting inflammation in CVD, while overwhelming evidence indicates that inflammation is a driver of chronic CVD as well as detrimental to repair processes following acute CV events, broad inhibition of these pathways using existing anti-inflammatories has so far not been successful. Future studies may aim to alter the timing of intervention, for example in MI recovery where initial inflammation is beneficial, or to investigate efficacy in just a subset of patients, such as in CANTOS where treatment was limited to patients whose inflammatory state was already elevated above baseline.

## 3 Emerging anti-inflammatory drug repurposing candidates and molecular targets for cardiovascular disease

Many repurposing-candidates with anti-inflammatory activity have been investigated in various *in vitro* assays and animal models of CVD with some promising results (Table 2). This new generation of repurposed anti-inflammatory drugs have largely been pursued for their activity against specific molecular targets and pathways known to be involved in CVD rather than for observations from other patient cohorts, as has previously been the case. In fact, now many drugs that have not been originally developed as anti-inflammatory medicines are being identified to have novel anti-inflammatory activity in drug repurposing screens.

**TABLE 2:** Drug repurposing candidates with an anti-inflammatory mode of action in cardiovascular disease models.

Pharmacological agent	Biological target	Original indication	Disease model	Key findings	References
Digoxin	ATPase, stimulates parasympathetic nervous system	Heart failure and arrhythmias	Diet-induced obesity in mice	Digoxin inhibits IL-17 A production, alleviates metabolic disorder, promotes glucose homeostasis, adipose-tissue browning, thermogenesis and energy expenditure	Teijeiro et al. (2021)
Disulfiram	Acetaldehyde dehydrogenase	Supports the treatment of chronic alcoholism	Diet-induced obesity in rats	Reduced body weight gain and decreased body weight of obese animals	Omran et al. (2020)
Flurbiprofen	Cyclooxygenase 1/2	Osteoarthritis, rheumatoid arthritis	Diet-induced obesity in mice	Reduced body weight after onset of obesity, visceral fat accumulation and leptin resistance	Hosoi et al. (2014)
Gefitinib	Epidermal Growth Factor Receptor (EGFR)	Non-small cell lung carcinoma	Diet-induced obesity in mice	Gefitinib lowers blood glucose in all mice and glucose-stimulated insulin secretion in Ripk2−/− mice	Duggan et al. (2020)
Ibrutinib	Bruton’s Tyrosine Kinase (BTK), NLRP3 inflammasome	X-linked agammaglobulinemia, mantle cell lymphoma, leukaemias	Diet-induced obesity in mice	Ibrutinib improves glycemic control, reduces myeloid cell recruitment to liver, adipose tissue and kidney. Decreases NF-kB and NLRP3 inflammasome activation	Purvis et al. (2020)
Imatinib	Tyrosine kinases e.g. c-Abl, PDGFR, c-Kit, c-Fms, Lck	Philadelphia chromosome^+^ leukaemias, gastrointestinal stromal tumors	Diet-induced obesity in mice	Imatinib lowers blood glucose in all mice and increases glucose-stimulated insulin secretion in Ripk2−/− mice	Duggan et al. (2020)
Lamivudine	Reverse transcriptase, NLRP3 inflammasome	HIV, Hepatitis B	Diet-induced obesity in mice	improves glucose tolerance and insulin sensitivity, inhibits inflammasome activity. Reverses insulin resistance in tissues of type 2 diabetic patients	Ambati et al. (2020)
Niclosamide	Inhibitor of oxidative phosphorylation and stimulator of ATPase activity in mitochondria	Helminth infection	*Ldlr* ^*−/−*^ high fat diet mouse model of atherosclerosis	Decreased aortic and carotid artery calcification, improved fatty liver features, decreased cholesterol levels. Inhibits Wnt/ß-catenin, mTORC1, STAT3, NF-kB and Notch signaling pathways	Tanaka et al. (2021)
Nitazoxanide	pyruvate:ferredoxin oxidoreductase (PFOR)	Protozoa, helminth, viral and bacterial infections	Peripheral Blood Mononuclear Cells (PBMCs) from patients with type 2 diabetes	Inhibits T cell proliferation, secretion of (IL)-1β, IL-2, IL-6, IL-10, and IL-12, decreases M1 and increases M2 macrophage population	Castillo-Salazar et al. (2021)

One notable molecular pathway and exciting therapeutic target that has been of particular research interest is the NLRP3 inflammasome. Pharmacological inhibition of the NLRP3 inflammasome by drugs such as Bruton’s tyrosine kinase inhibitors (Ito et al., 2015; Purvis et al., 2020) has shown benefit in pre-clinical models of CVD. Moreover, success in trails with IL-1 inhibitors and colchicine is thought to be predominantly due to effects on the inflammasome pathway, and so repurposed inflammasome inhibitors could hold great potential for future CVD treatment.

Moreover, with this new wave of repurposing candidates the opportunity exists to look for drugs which actively enhance the processes of inflammation resolution and tissue repair, rather than simply suppress detrimental inflammation. This is a highly active area of research (Panigrahy et al., 2021), and novel therapeutics such as the specialised pro-resolving mediator aspirin-triggered lipoxin-A4 have shown promise in pre-clinical animal models of CVD (Petri et al., 2017). It remains to be seen whether enhancement of inflammation resolution will be achievable in clinical trials, and indeed whether any repurposed drugs are capable of inducing inflammation resolution and tissue repair in CVD.

## 4 Future directions for drug repurposing in cardiovascular inflammation

In this section we highlight recent findings from proof of concept and pre-clinical studies that offer new avenues for drug repurposing in CVD.

### 4.1 Drug re-engineering and modified drug delivery systems

Drug re-engineering and engineering drug delivery systems are rapidly gaining popularity alongside conventional drug repurposing. The aim is to enhance aspects of drug efficacy, for instance by improving pharmacokinetics and limiting off-target effects, and this is achieved through subtle alteration of chemical structures or repackaging drugs inside specialised delivery vehicles. Examples of engineered drug delivery systems are highly varied, with popular options including liposomes, exosomes, and nanotechnology-based approaches (Suri et al., 2007; van der Koog et al., 2022), and many of these are already being explored for delivery of anti-inflammatory medicines (Wang et al., 2021). While these approaches are not strictly drug repurposing and will require additional testing to ensure any modifications are safe and well tolerated, these strategies based upon existing drugs do reduce risk of failure from drug inefficacy, making them still favourable approaches in the eyes of pharmaceutical companies.

### 4.2 Macrophage-targeted drug delivery methods

As key immune cells in the pathology of CVD, macrophages are a major target for emerging therapeutics and have been of particular focus of drug re-engineering strategies to specifically target drug delivery. At its best, drug re-engineering has the potential to achieve more than simply limiting undesirable side-effects and should ideally aim to actively confer additional therapeutic benefits. An example of this is the use of phosphatidylserine (PS) containing liposomes as a delivery system. PS is highly abundant on the surface of apoptotic cells and is a key “eat me” molecule for the recognition of apoptotic cells by macrophages during the important process of efferocytosis in inflammation resolution. PS-containing liposomes are therefore able to act as apoptotic cell mimetics. In their 2019 study, Wu et al. used these apoptotic cell mimetics to package a PPARγ agonist in an *ApoE* ^*−/−*^ murine model of atherosclerosis, in which they were able to confirm that the constructs are effectively targeted to macrophages within atherosclerotic plaques (Wu et al., 2019). This strategy has the dual benefit of allowing targeted delivery of the chosen drug to macrophages, plus inducing anti-inflammatory and pro-repair signalling pathways, such as IL-10 production, which are known to be triggered by efferocytosis (Gordon and Plüddemann, 2018).

Similarly, a recent series of studies by researchers at the University of Illinois synthesised forms of dexamethasone bound to a nanocarrier of high-molecular weight dextran. Dexamethasone is a classic corticosteroid that has been used for the treatment of RA since its first synthesis in 1957 by Philip Showalter Hench (Arth et al., 1958) and it is now one of the most widely used anti-inflammatory medicines on the market. Furthermore, in June 2020 dexamethasone was identified as an effective repurposed drug for the treatment for life-threatening lung inflammation associated with COVID-19 infection in the landmark RECOVERY trial (2021), leading to a resurgence of interest in this classic anti-inflammatory drug. Dexamethasone exerts its anti-inflammatory effects *via* suppression of the hypothalamic-pituitary-adrenal (HPA) axis as well as by binding of the glucocorticoid receptor (GR) to influence gene transcription. As GR is expressed in many cell types, dexamethasone is non-specific in its activity and consequently has adverse off-target effects with long-term use. Through a drug re-engineering approach, Ma et al. (2016) aimed to mitigate these side effects by selectively targeting phagocytic immune cells. In the 2016 study, the authors show injection of lean and obese C57BL/6J mice with dextran conjugates results in preferential distribution to the visceral adipose tissue (VAT) of obese mice when delivered intraperitoneally (i.p.) but not intravenously. Having optimised the size of the dextran conjugates the authors demonstrated that dextran chemically linked to dexamethasone delivered intraperitoneally significantly reduced expression of mRNA encoding the inflammatory mediators TNFα, IL-6 and CCL2 in four different adipose tissues of obese mice. The authors proposed that their re-engineered forms of dexamethasone were able to selectively target drugs to macrophages and suggested that this is achieved *via* binding to macrophage-expressed mannose and scavenger receptors. However, a critical test of the mechanism of targeting adipose resident macrophages has not been reported.

In a recent follow up paper, Prabhu et al. (2021) used the same re-engineered forms of dexamethasone in longer term pre-clinical studies to investigate if they could ameliorate metabolic dysfunction and systemic inflammation. Importantly, the authors performed a direct comparison of nanocarrier-dexamethasone (ND) with the same concentration of free dexamethasone. In pilot experiments, the authors confirmed that they were able to reproduce specific targeting of macrophages in the adipose tissue of a mouse model of obesity to reduce production inflammatory gene expression and cytokine production. Moreover, the authors showed that nanocarrier dextran, but not similar concentrations of free dexamethasone, could induce lipolysis from adipose tissue. Remarkably, in a 35-day study the authors showed that daily i. p. Delivery of ND resulted in a 20% reduced body weight (Prabhu et al., 2021). These exciting findings exemplify the potential of drug re-engineering adding benefit beyond reducing off-target effects.

It is important to note that in these studies not only was a steroidal anti-inflammatory drug re-engineered into a nanocarrier format, but the route of drug delivery was important for therapeutic efficacy *in vivo*, namely intraperitoneal *versus* intravenous delivery. Intraperitoneal delivery is used only in exceptional circumstances clinically, for instance in treatment of ovarian or pancreatic cancer, and the authors speculate that intraperitoneal delivery of a slow-release form of re-engineered dexamethasone (and indeed other repurposed anti-inflammatory drugs) could be performed several times a year by a trained medical professional (Ma et al., 2016), although oral delivery remains the preferable drug administration route.

### 4.3 Optimising dosing schedules (chrono-pharmacology)

Chrono-pharmacology refers to the dosing of drugs in a time-specific manner in order to optimise drug efficacy. In a seminal paper published in 2014, Zhang et al. generated a circadian gene atlas in mammalian tissues using RNA-Sequencing (RNASeq) and DNA arrays to study the transcriptomes of 12 different tissues every 2 h. Remarkably, 43% of mouse protein coding genes were shown to be regulated in a circadian manner in at least one organ. It had been known that endogenous 24-hour oscillations in biological signalling systems regulated many important physiological processes but Zhang et al. focused attention on the possibility that therapeutic targets for current and future therapies are regulated in a circadian manner. They note that 56 of the top 100 best-selling drugs in the USA targeted genes with rhythmic expression and more than 50% of these drugs have a half-life of less than 6 h, suggesting that the timing of drug delivery could be an underappreciated aspect of drug efficacy (FitzGerald, 2014; Zhang et al., 2014). Indeed, this phenomenon has previously been noted in CVD with use of statins, which are best taken before bedtime due to their short half-life coupled with increased cholesterol production at night (Awad and Banach, 2018). Furthermore, CV events are known to have strong circadian incidence with both stroke and MI being more prevalent in the morning, while chronic circadian disruption through shift work is associated with increased CV risk (Thosar et al., 2018). Moreover, the immune system is under direct circadian control with oscillating numbers of circulating immune cells and cyclic expression of immune mediators such as cytokines TNFα and IL-1β (Scheiermann et al., 2013). This is observable in patients with autoimmune disease who often experience periodicity in the severity and onset of their symptoms (Scheiermann et al., 2013; Torres-Ruiz et al., 2017).

The emerging concept of chrono-pharmacology aims to utilise this natural phenomenon in order to optimise drug efficacy. The best example of the potential for chrono-pharmacology in CV inflammation comes from the work of Scheiermann and colleagues (Winter et al., 2018). In their 2018 paper, Winter et al. explored the diurnal regulation of leukocyte adhesion to atherosclerotic lesions in major arteries. The authors maintained male *Cx*_*3*_ *cr* ^*GFP/WT*^ *, ApoE* ^*−/−*^ mice on a high fat diet on a strict 12-hour light dark cycle [lights on = Zeitgeber Time 0 (ZT0)]. Classical monocyte and neutrophil numbers in blood, bone marrow and spleen were found to oscillate, whereby levels peaked at ZT21-ZT1 with around a two-fold variance over the 24-hour period. Using *in vivo* microscopy, the authors extended their studies to analysing monocyte and neutrophil adhesion to the carotid bifurcation and revealed rhythmic adhesion peaking at ZT1 and a dipping at ZT13, persisting even in the absence of light cues*.*

Winter et al. (2018) implicated CCL2-CCR2 chemokine signalling in this diurnal pattern of leukocyte recruitment by studying oscillation in the plasma levels of chemokines previously shown to be important in leukocyte rolling and tight adhesion to activated endothelial cells. Levels of CCL2 but not CCL5 or CX_3_CL1 chemokines were higher in plasma of hypercholesterolemic mice at ZT1 than ZT13 and circulating monocytes and neutrophils were identified as potential sources of circulating CCL2. This periodicity in CCL2 plasma expression was subsequently mapped to the clock protein BMAL1 through specific deletion in aortic endothelial cells or myeloid cells. The authors then demonstrated this mechanism *ex vivo* using adhesion assays involving adhesion of monocytes to activated endothelial cells in the presence of plasma obtained at ZT1 and ZT13 and confirmed the leading role of CCL2 levels using neutralising CCR2 blocking antibodies.

Despite this technical *tour de force,* perhaps the most impressive observation was demonstration of chrono-pharmacology in long-term experimental models. In an *ApoE* ^*−/−*^ high-fat diet murine model of atherosclerosis, delivery of a small molecule CCR2 antagonist showed significant decrease in plaque size and lesional macrophage numbers only in mice treated at ZT17 but not when the same antagonist was applied at ZT5. The same chrono-pharmacological blockade of CCR2 was applied to a second model of inflammatory disease, LPS-induced lung injury, where myeloid cell recruitment was again seen only with drug delivery at ZT13 but not ZT1, fully demonstrating the power of chrono-pharmacology. The CCR2-CCL2 axis has not yet been successfully targeted in CV inflammation, despite striking results in pre-clinical models (Georgakis et al., 2022). Sophisticated therapeutic approaches such as that used by Winter et al. (2018) could be the key targeting such inflammatory pathways where previous attempts have failed.

## 5 Discussion

One of the greatest challenges currently faced by targeting inflammation in CVD or any chronic inflammatory disease is the fact that the very same inflammatory processes can be both beneficial and pathological, depending on duration and context. For instance, the components of inflammatory pathways that are major therapeutic targets like NF-κB, TNFα, and the NLRP3 inflammasome are shared across many cell types and mediate cellular processes from cell survival to host defence against infection to chronic inflammatory disease. As a consequence, it is inherently not possible to target such pathways only in a pathological context without risking disruption to overall functional immunity and tissue homeostasis. This principle is exemplified in clinical trials of single cytokine inhibiting therapies such as IL-6 inhibitors in patients with myocardial infarction (Ritschel et al., 2014). Despite IL-6 signalling being generally considered pro-inflammatory and therefore injurious, lack of benefit in clinical trials suggests that these very same pro-inflammatory pathways may play an important role in early repair. Moreover, broad and prolonged inhibition of pro-inflammatory pathways is inevitably detrimental even in patients with chronic inflammation and CVD, as is exemplified in CV trials of IL-1 inhibitors and colchicine where the risk of serious infection and sepsis is increased (Ridker et al., 2017; Tardif et al., 2019; Tong et al., 2020).

Despite this, feasibility for therapeutic interventions to treat cardiovascular inflammation lies within the subtle variations to how important certain inflammatory pathways are in specific disease contexts or at particular disease stages. For instance, monocyte recruitment is a crucial process in early atherogenesis and so therapies which interfere with this process, such as those which may inhibit chemokine signalling or cytoskeletal rearrangements, may be beneficial in patients with early-stage disease, although identifying populations with subclinical disease remains a challenge. Conversely, at advanced disease stages where the largest pathological contribution shifts from monocyte recruitment towards local proliferation within lesions (Libby, 2021), these interventions would be less appropriate. Prevention of long-term immunosuppression is possibly the largest obstacle to overcome in the development of anti-inflammatory medicines for CVD, so therefore inflammation associated with acute cardiovascular injury may be a more suitable clinical application for future anti-inflammatory therapies, rather than the prevention or management of chronic disease.

Given the current productivity crisis in the pharmaceutical industry, a practical solution is to innovate strategies that make existing anti-inflammatory drugs better suited to long-term use in the clinic, rather than to generate novel drugs that are prone to the same shortcomings as currently available medicines. Drug repurposing and related approaches such as drug re-engineering or adapting drug delivery methods provide a way to address this need, and pre-clinical data have so far been promising. The concept of chrono-pharmacology complements this idea and may allow for reconsideration of failed drugs with undesirable pharmacokinetics, as these effects especially a short half-life may in fact confer fewer adverse drug effects.

An exciting new generation of drug re-purposing candidates is emerging based on mechanistic insights, with many of these medicines being borrowed from unrelated medical fields and with their anti-inflammatory activity only recently being identified. In the future, high-throughput screening and target-based repurposing approaches will surely reveal more candidates such as these that may prove to be of clinical use. Despite the perhaps inevitable shift to systematic drug repurposing approaches however, there remains much untapped knowledge on routinely used clinical medicines that may be helpful in uncovering new candidates for CVD treatment. A combination of patient data meta analyses and sub-analyses of non-cardiovascular clinical trials could allow data mining to reveal such patterns.
